# INHBB Is a Novel Prognostic Biomarker Associated with Cancer-Promoting Pathways in Colorectal Cancer

**DOI:** 10.1155/2020/6909672

**Published:** 2020-10-06

**Authors:** Jinpeng Yuan, Aosi Xie, Qiangjian Cao, Xinxin Li, Juntian Chen

**Affiliations:** ^1^Department of Gastrointestinal Surgery, The First Affiliated Hospital of Shantou University Medical College, Shantou, 515041 Guangdong, China; ^2^Shantou University Medical College, China

## Abstract

**Background:**

Inhibin subunit beta B (INHBB) is a protein-coding gene that participated in the synthesis of the transforming growth factor-*β* (TGF-*β*) family members. The study is aimed at exploring the clinical significance of INHBB in patients with colorectal cancer (CRC) by bioinformatics analysis.

**Methods:**

Real-time PCR and analyses of Oncomine, Gene Expression Omnibus (GEO), and The Cancer Genome Atlas (TCGA) databases were utilized to evaluate the INHBB gene transcription level of colorectal cancer (CRC) tissue. We evaluated the INHBB methylation level and the relationship between expression and methylation levels of CpG islands in CRC tissue. The corresponding clinical data were obtained to further explore the association of INHBB with clinical and survival features. In addition, Gene Set Enrichment Analysis (GSEA) was performed to explore the gene ontology and signaling pathways of INHBB involved.

**Results:**

INHBB expression was elevated in CRC tissue. Although the promoter of INHBB was hypermethylated in CRC, methylation did not ultimately correlate with the expression of INHBB. Overexpression of INHBB was significantly and positively associated with invasion depth, distant metastasis, and TNM stage. Cox regression analyses and Kaplan-Meier survival analysis indicated that high expression of INHBB was correlated with worse overall survival (OS) and disease-free survival (DFS). GSEA showed that INHBB was closely correlated with 5 cancer-promoting signaling pathways including the Hedgehog signaling pathway, ECM receptor interaction, TGF-*β* signaling pathway, focal adhesion, and pathway in cancer. INHBB expression significantly promoted macrophage infiltration and inhibited memory T cell, mast cell, and dendritic cell infiltration. INHBB expression was positively correlated with stromal and immune scores of CRC samples.

**Conclusion:**

INHBB might be a potential prognostic biomarker and a novel therapeutic target for CRC.

## 1. Introduction

Colorectal cancer (CRC) is the fourth most common cause of cancer-related death, causing at least 90,000 deaths every year, and its morbidity has increased yearly [1]. Although more and more molecular mechanisms have been delineated in the tumorigenesis and metastasis of CRC, the overall survival of patients remains low, especially in stage III-IV patients [2, 3]. This is mainly because of delayed diagnosis and treatment, metastasis before treatment, and recurrence after surgery [4, 5]. Consequently, it is essential to further explore pathogenesis and metastasis of colorectal carcinoma and to find the potential prognostic biomarkers for early diagnosis and therapy.

The inhibin subunit beta B gene (INHBB) encodes a preprotein that is proteolytically processed to inhibin and activin, functional cytokines belonging to the transforming growth factor-*β* (TGF-*β*) family [6, 7]. Among them, activin B is a homodimer of two subunits encoded by INHBB. Recently, INHBB has been considered a novel oncogene in various cancer types. Kita et al. indicated that high-level expression of INHBB is correlated with regional lymph node metastasis in oral cancer and promotes cell proliferation and migration [8]. Wijayarathna and de Kretser suggested that overexpression of activin B promotes tumor progression of reproductive organs [9]. Furthermore, Tamminen et al. indicated that elevated expression of activin B promotes mesothelioma cell invasion and migration by activating the ERK/Smad2/3 pathway [10]. These studies indicated that INHBB might play essential roles in tumorigenesis and migration. However, few studies evaluated the relationship between INHBB and clinical features in patients with colorectal cancer, especially for prognosis.

In the current study, we aim to detect the expression level of INHBB in colorectal cancer compared to adjacent normal tissue by utilizing the Gene Expression Omnibus (GEO) and The Cancer Genome Atlas (TCGA) databases and further investigate the association between INHBB expression and clinical features. Univariate and multivariate Cox analyses and Kaplan-Meier survival analysis were performed to explore the prognostic value. Additionally, Gene Set Enrichment Analysis (GSEA) was performed to evaluate the gene ontology (GO) and signaling pathways of INHBB involved in colorectal cancer.

## 2. Material and Methods

### 2.1. Tumor Samples

The fresh primary tumor specimens and their corresponding adjacent nontumor tissues were obtained from colorectal cancer patients who underwent surgery at the First Affiliated Hospital of Shantou University Medical College in 2020. The specimens were stored at −80°C immediately after surgery. This study was approved by the Institutional Research Ethics Committee of the First Affiliated Hospital of Shantou University Medical College. All patients who participated in the study signed informed consent.

### 2.2. Data Acquisition

We ascertained the transcript level of INHBB expression in various cancers by the Oncomine database (https://www.oncomine.org/resource/main.html) using a top gene rank 10%, fold change > 2, and *P* value < 1*E* − 4 as the thresholds. The gene expression profiles and DNA methylation data with their corresponding clinical data were downloaded from COAD and READ projects in TCGA database, and microarray data of CRC was downloaded from the GEO database. Among them, TCGA, GSE39582, and GSE38832 datasets with survival data were further analyzed for the prognostic value of INHBB. TCGA expression and four GEO datasets (GSE23878, GSE44076, GSE25070, and GSE44861) with normal colon and CRC tumor tissues were analyzed for the differential expression of INHBB. The primary information of TCGA and GEO datasets is shown in Table 1.

### 2.3. Statistical Analysis

R software was utilized for statistical analysis. The Wilcoxon test was used to evaluate the expression and methylation level of INHBB between CRC tissue and normal tissue in the GEO (GSE23878, GSE44076, GSE25070, and GSE44861) and TCGA CRC datasets. And the Spearman correlation analysis was performed to explore the relationship between INHBB expression and CpG island methylation, and the relationship between INHBB and clinicopathologic features was evaluated by using chi-squared and logistic regression tests in TCGA patients. The prognostic value was evaluated by the Kaplan-Meier method in TCGA cohort and further validated in the GSE38832 and GSE39582 datasets. Also, we performed univariate and multivariate Cox regression analyses to explore the independent prognostic value of INHBB expression. *P* < 0.05 was considered statistically significant in the above analyses.

### 2.4. Gene Set Enrichment Analysis

GSEA is a new computational method to evaluate whether a predetermined set of genes show statistically significant differences between two different biological states [11]. GSEA 4.1 software was used to explore the biological functions of INHBB expression in CRC. Patients in TCGA CRC dataset were divided into two groups (high-expression group and low-expression group) according to the expression level of INHBB. We downloaded the relevant datasets from the Molecular Signatures Database [12, 13] (MsigDB, https://www.gseamsigdb.org/gsea/msigdb/) to analyze GO terms and KEGG pathways to investigate the potential function of INHBB, basing the analyses on default weighted enrichment statistics, repeating the analysis 2000 times at a time. FDR *q*‐value < 0.05 and NES > 1.0 were set as the cut-off criteria.

### 2.5. Quantitative Real-Time Polymerase Chain Reaction

RNA extraction from CRC or adjacent tissue samples was performed using the TRIzol reagent (Thermo Fisher Scientific). The cDNA was generated by using the Geneseed® II First Strand cDNA Synthesis Kit. Complementary DNA primers specific for INHBB amplification were as follows: forward, 5′-CCTGAAACTCCTGCCCTACG-3′, and reverse, 5′-CCACCATGTTCCACCTGTCA-3′. In a 20 *μ*L reaction system, the qPCR was performed according to the instructions. Ten-microliter 2xqPCR SYBR-Green Master Mix (Vazyme Biotech), 0.4 *μ*L forward primer (10 *μ*M), 0.4 *μ*L reverse primer (10 *μ*M), and 5 *μ*L cDNA were included in the 20 *μ*L reaction system. All specimens were tested in triplicate. Relative mRNA levels of INHBB were normalized to GAPDH expression.

## 3. Results

### 3.1. Exploration of the INHBB Expression and Methylation Status in CRC

**Figure 1:**
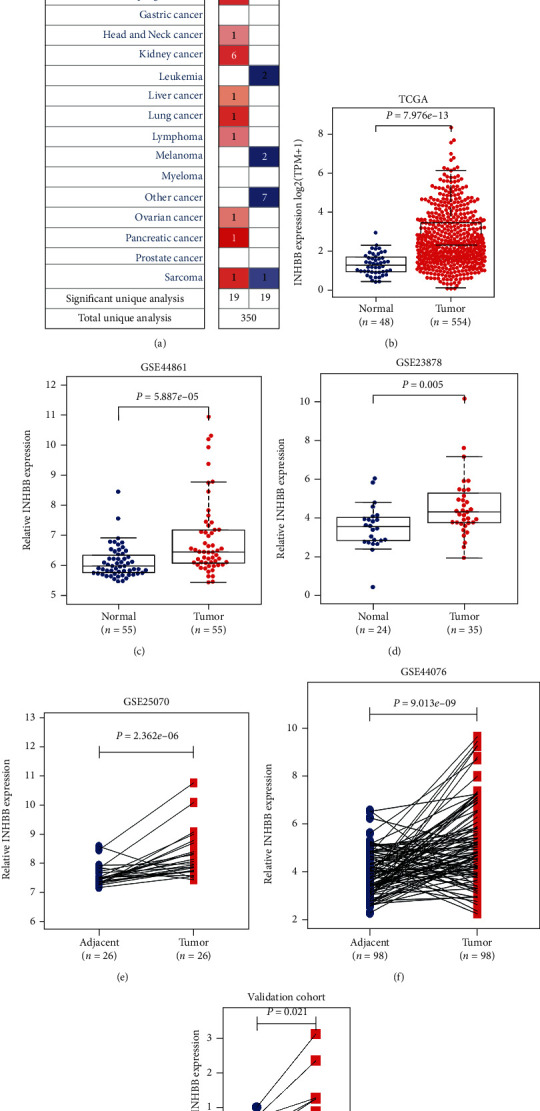
INHBB expression in colorectal cancer tissue and adjacent normal tissue. (a) The expression level of INHBB in cancers in the Oncomine database: the left box in red indicated the number of datasets with high expression of INHBB and the right box in blue indicated the number of datasets with low expression of INHBB after comparing cancerous and normal tissues. (b–g) TCGA cohort, GSE23878, GSE44076, GSE25070, and GSE4486 from the GEO database and our validation cohort indicated that INHBB was highly expressed in colorectal cancer.

**Figure 2:**
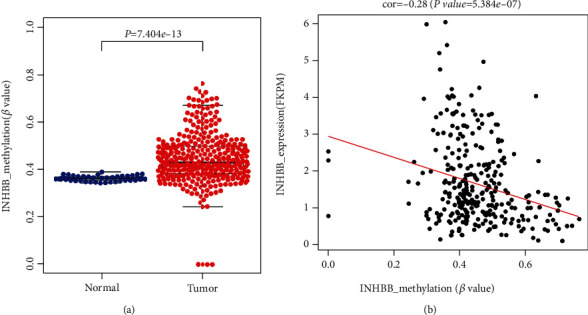
The relationship between INHBB expression and methylation. (a) INHBB was hypermethylated in colorectal cancer compared to adjacent normal tissue (*P* < 0.001), and (b) INHBB expression was negatively correlated with promoter methylation (Cor: -0.28, *P* < 0.001).

INHBB expression was elevated in various cancers, such as brain cancer, colorectal cancer, esophageal cancer, head and neck cancer, and kidney cancer (Figure 1(a)). However, INHBB was downregulated in some cancers, such as breast cancer, cervical cancer, and leukemia. We further explored the INHBB expression in paired or unpaired adjacent normal and tumor samples by analyzing TCGA and GEO datasets and validated it in our cohort. INHBB was highly expressed in TCGA, GSE23878, GSE44076, GSE25070, and GSE44861 datasets and the validation cohort (Figures 1(b)–1(g)). To explore the relationship between INHBB promoter methylations and expression, the methylation data including 25 CpG sites were downloaded from TCGA database. We explored the promoter methylation differences of INHBB between CRC and normal control and found that CRC tissue had a higher methylation level of INHBB compared to normal control in TCGA database (Figure 2(a)). Furthermore, the methylation differences of 25 CpG sites between CRC tissue and normal control were assessed, and the result indicated that 18 of them were highly methylated and 7 were lowly methylated in CRC tissue (Supplementary Table 1). It has been previously reported that the promoter methylation level is negatively correlated with gene expression level [14]. We explored the relationship between INHBB expression and promoter methylation, and the results indicated that INHBB expression was negatively correlated with promoter methylation (Figure 2(b); Cor: -0.28, *P* < 0.001). In addition, the relationship between INHBB expression and methylations of these 25 CpG sites was explored, and the results are shown in Supplementary Table 2. After screening these results, we cannot find the key methylation sites which play essential roles in the regulation of INHBB expression for the low correlation and mismatch between CpG site methylation level and INHBB expression. Thus, these results suggest that promoter methylation may not be the main mechanism to regulate INHBB expression.

### 3.2. Association with INHBB Expression and Clinicopathologic Characteristics in TCGA Cohort

High expression of INHBB was closely related to invasion depth (*P* < 0.001), lymph node metastasis (*P* < 0.001), distant metastasis (*P* < 0.001), and TNM stage (*P* < 0.001) (Table 2). Other clinical features, such as gender and age, were not correlated with INHBB expression. Univariate logistic regression analysis was conducted to evaluate the relationship between INHBB expression and these clinicopathologic variables (Table 3). Overexpression of INHBB was significantly and positively associated with invasion depth (T3 and T4 vs. T1 and T2, OR: 1.78, 95% CI: 1.16-2.75, *P* = 0.008), lymph node metastasis (yes vs. no, OR: 1.80, 95% CI: 1.27-2.55, *P* < 0.001), distant metastasis (yes vs. no, OR: 1.76, 95% CI: 1.08-2.91, *P* = 0.026), and TNM stage (stages III and IV vs. stages I and II, OR: 1.63, 95% CI: 1.16-2.31, *P* = 0.005). Other variables, such as gender (female vs. male, OR: 1.17, 95% CI: 0.83-1.63, *P* = 0.364) and age (>65 vs. <65, OR: 0.93, 95% CI: 0.66-1.32, *P* = 0.700), showed no significant difference. Altogether, these results indicated that INHBB may function as an oncogene in CRC, and patients with INHBB overexpression were more likely to progress to tumor metastasis, worse invasion depth, and TNM stage.

### 3.3. Prognostic Value of INHBB Expression in CRC

**Figure 3:**
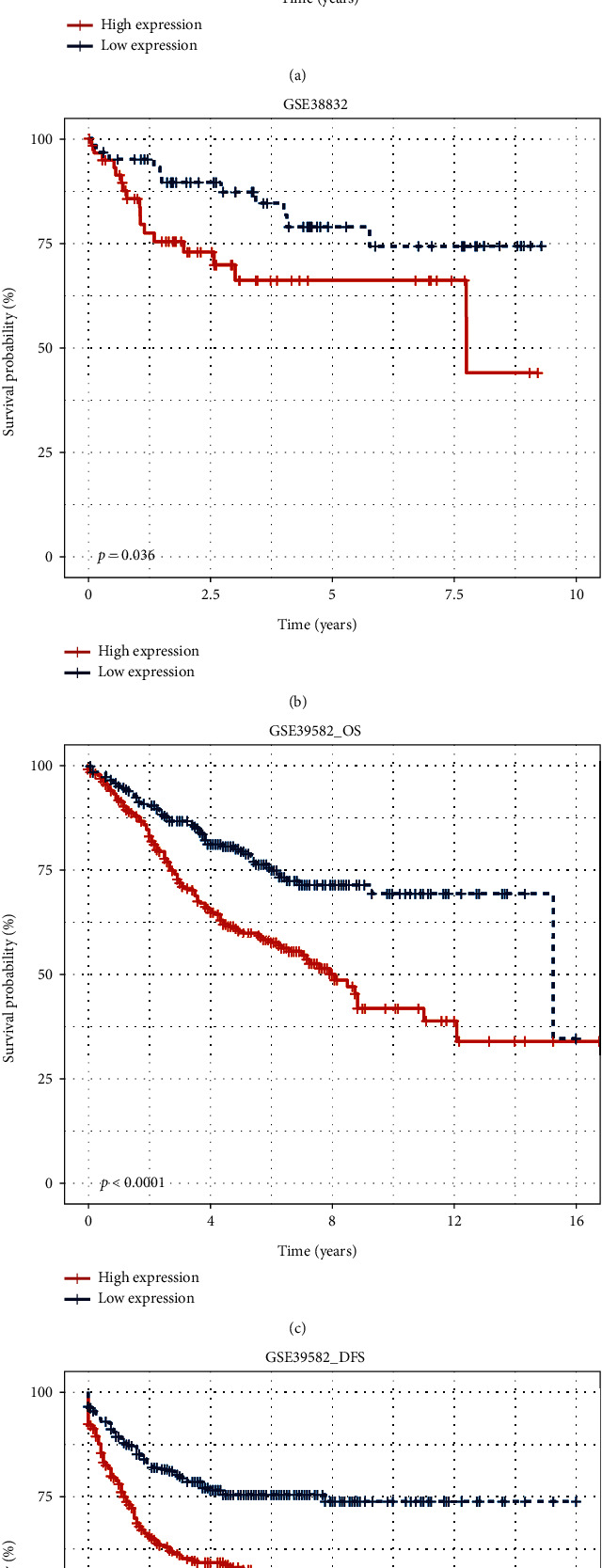
The prognosis value of INHBB expression for the OS and DFS of colorectal cancer in TCGA cohort and GEO datasets. High expression of INHBB indicated poor survival in (a) TCGA (OS, *P* < 0.001), (b) GSE38832 (OS, *P* = 0.036), (c) GSE39582 (OS, *P* < 0.001), and (d) GSE39582 (DFS, *P* < 0.001).

First, we performed the Kaplan-Meier survival analysis on TCGA CRC dataset (Figure 3(a)) to evaluate the prognostic value of INHBB and then validated the prognostic value in several GEO datasets. The results indicated that high expression of INHBB was significantly associated with worse OS in TCGA CRC (Figure 3(a), *P* < 0.001), GSE38832 (Figure 3(b), *P* = 0.036), and GSE39582 (Figure 3(c), *P* < 0.001) datasets. Elevated expression of INHBB was significantly associated with DFS in the GSE39582 dataset (Figure 3(d), *P* < 0.001). To further explore the independent prognostic value, univariate and multivariate Cox regression analyses were performed using TCGA CRC patients (Table 4). Univariate analysis demonstrated that high expression of INHBB (*P* < 0.001), age (*P* = 0.002), invasion depth (*P* < 0.001), lymph node metastasis (*P* < 0.001), distant metastasis (*P* < 0.001), and TNM stage (*P* < 0.001) corresponded with poor OS in CRC patients. Multivariate analysis demonstrated that high expression of INHBB (*P* = 0.039), age (*P* < 0.001), invasion depth (*P* = 0.002), distant metastasis (*P* = 0.006), and TNM stage (*P* = 0.014) also corresponded with poor OS in CRC patients. Hence, INHBB expression, age, distant metastasis, invasion depth, and TNM stage were independent prognostic factors for OS in CRC.

### 3.4. GO and KEGG Pathway Enrichment Analysis by GSEA

**Figure 4:**
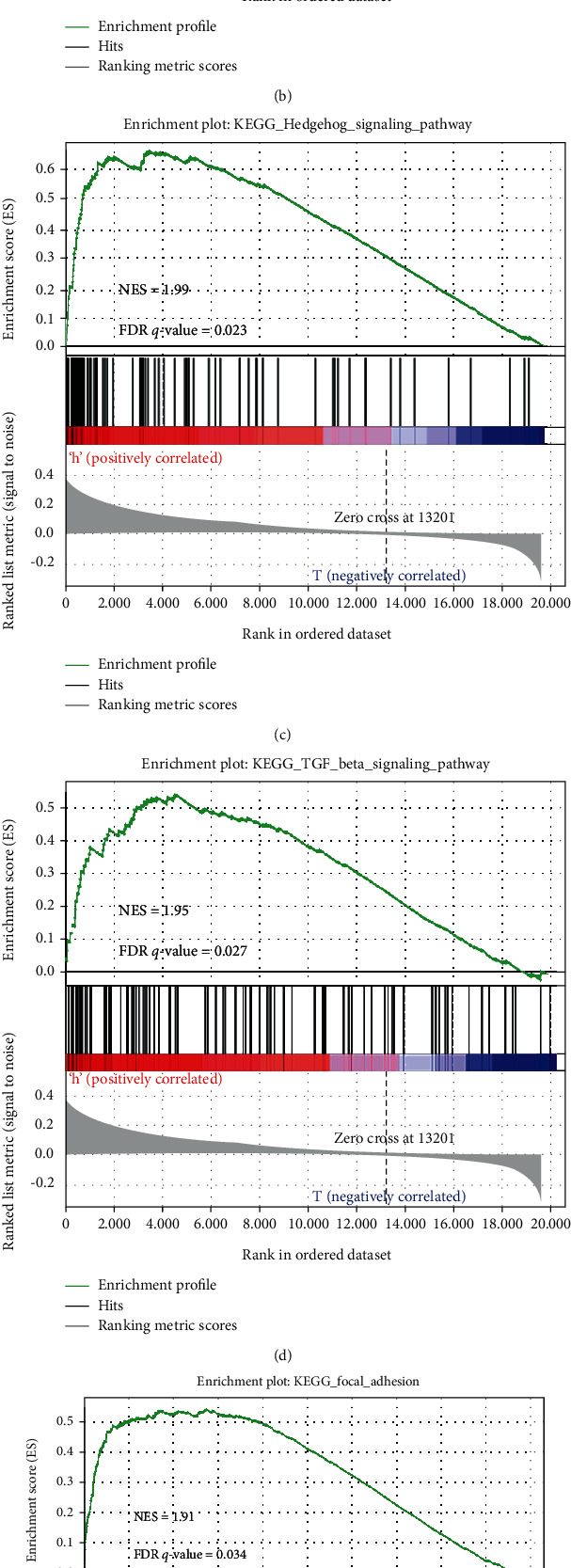
GO and KEGG pathway enrichment analysis by GSEA. (a) Top 5 GO cellular components, biological process, and molecular functions that were enriched in the INHBB high-expression group. (b–f) Five cancer-promoting signaling pathways enriched in the INHBB high-expression group, including the Hedgehog signaling pathway, ECM receptor interaction, TGF-*β* signaling pathway, focal adhesion, and pathway in cancer. (g) Leading edge analysis of GSEA evaluations was performed on the INHBB gene: (i) ECM receptor interaction, (ii) focal adhesion, (iii) TGF-*β* signaling pathway, (iv) Hedgehog signaling pathway, and (v) pathway in cancer.

Gene Ontology contains cellular components, biological process, and molecular function. The top 5 for each category are shown in Figure 4(a). The enriched cellular components were the basement membrane, filopodium, I band, distal axon, and axon part. The enriched biological processes were cell volume homeostasis, morphogenesis, mesenchyme development, heart morphogenesis, and mesenchymal cell differentiation. The enriched molecular functions were extracellular matrix, scaffold protein binding, growth factor binding, collagen binding, and actin binding. A total of 11 signaling pathways were enriched in the INHBB high-expression group (Table 5), and elevated expression of INHBB was positively associated with the Hedgehog signaling pathway, ECM receptor interaction, TGF-*β* signaling pathway, focal adhesion, and pathway in cancer (Figures 4(b)–4(f)). Pathways in cancer contain the regulation of many cancer-promoting pathways including these 4 signaling pathways, which suggests that INHBB regulates CRC by mediating these cancer-promoting pathways. We further performed leading edge analysis and found a close association between INHBB and these cancer-promoting pathways (Figure 4(g)).

## 4. Discussion

INHBB is a subunit of the homodimer activin B, which is a member of the TGF-*β* superfamily of cytokines [9]. INHBB or activin B has been reported to be overexpressed in various malignant tumors and plays essential roles in tumor proliferation, invasion, and migration, such as oral cancer [8], endometrial cancer [15–17], prostate cancer [18, 19], renal clear cell carcinoma [20], and thyroid cancer [21]. INHBB has been reported to be significantly associated with the prognosis of many tumors. Therefore, INHBB might be an oncogene and play roles in tumor proliferation, invasion, and migration. However, the functions of INHBB have not been reported in CRC.

In the present study, we first found that INHBB is highly expressed in colorectal cancer compared to normal adjacent tissue by analysis of data in TCGA and GEO databases and finally validated it in our cohort. The promoter of INHBB was hypermethylated in CRC, and INHBB expression was negatively correlated with methylation. Furthermore, the overexpression of INHBB is significantly and positively associated with invasion depth, distant metastasis, and TNM stage. The Kaplan-Meier survival analysis suggested that high expression of INHBB is correlated with worse OS and DFS. In addition, univariate and multivariate analyses indicated that INHBB might be a novel independent prognostic factor in CRC. INHBB expression significantly promoted macrophage infiltration and inhibited memory T cell, mast cell, and dendritic cell infiltration. INHBB expression was positively correlated with stromal and immune scores of CRC samples.

In recent years, only some studies research the potential mechanisms of elevated INHBB expression in colorectal cancer and have shown that hypermethylation of the INHBB promoter is found in colorectal cancer [22, 23]. However, whether hypermethylation of INHBB ultimately affects its expression has not been explored in this research. Because promoter hypermethylation may influence gene expression [24], we further explored the methylation status and the relationship between INHBB expression and methylation in TCGA cohort and found that the promoter of INHBB is hypermethylated in CRC and INHBB expression is negatively correlated with methylation. This result indicated that the regulation of INHBB promoter methylation is not the main route to regulate INHBB expression in colorectal cancer. These results are consistent with Jiang and Hermeking who reported that the p53 gene is downregulated to silence miR-34, which eventually leads to the overexpression of INHBB in CRC [25]. We believe that many mechanisms for regulating INHBB expression remain undiscovered.

The mechanisms of INHBB in colorectal cancer have been rarely explored. INHBB and activin B are highly expressed in endometrial cancer, and activin B promotes cell adhesion, migration, and invasion via the SMAD2/3/integrin *β*3 signaling pathway [15]. Kita et al. suggested that INHBB and activin B are upregulated in oral cancer. And INHBB promotes cell migration and invasion via the EMT/activin B signaling pathway [8]. Hence, the INHBB gene might be involved in regulatory mechanisms of cancer through its encoded protein—activin B. Our study shows that high expression of INHBB is positively correlated with the Hedgehog signaling pathway, ECM receptor interaction, TGF-*β* signaling pathway, focal adhesion, and pathway in cancer which play essential roles in carcinogenesis and metastasis of CRC. Focal adhesion pathways have been reported to regulate the growth and invasion of CRC cells [26–28]. The Hedgehog pathway may alter the cell malignancy through EMT, gene mutation, metastasis, and angiogenesis in CRC [29–31]. The TGF-*β* signaling pathway is closely related to DNA damage response and DNA damage repair in CRC [32–34]. Leading edge analysis showed that INHBB is closely associated with these signaling pathways, indicating that INHBB or activin B might be involved in the regulation of colorectal cancer by mediating these cancer-promoting pathways and their downstream targets. Therefore, we have reasons to believe that INHBB might be a novel biomarker and therapeutic target for CRC.

## 5. Conclusion

In conclusion, this study is the first to describe the association between INHBB expression and its clinical features in CRC, especially its prognosis value. INHBB is overexpressed in CRC and associated with invasion depth, lymph node metastasis, distant metastasis, and TNM stage. Overexpression of INHBB is positively correlated with poor OS and DFS, and INHBB is an independent prognostic factor for colorectal cancer. INHBB may be a novel target for individualized treatment of colorectal cancer.

## Figures and Tables

**Table 1:** The basic information of TCGA and GEO datasets in the study.

Datasets	Data type	Platforms	Sample type	
			Tumor	Normal
TCGA	mRNA	Illumina HiSeq	554	48
TCGA	Methylation (*β* value)	Illumina HumanMethylation450	360	42
GSE23878	mRNA	Affymetrix Human Genome U133 Plus 2.0 Array	35	24
GSE25070	mRNA	Illumina HumanRef-8 v3.0 expression bead chip	26	26
GSE44076	mRNA	Affymetrix Human Genome U219 Array	98	98
GSE44861	mRNA	Affymetrix HT Human Genome U133A Array	55	55
GSE38832	mRNA	Affymetrix Human Genome U133 Plus 2.0 Array	122	—
GSE39582	mRNA	Affymetrix Human Genome U133 Plus 2.0 Array	579	—

**Table 2:** Association with INHBB expression and clinicopathologic characteristics in TCGA cohort.

	Total	Low expression	High expression	*χ* ^2^	*P*
Age					
Female	247	121	126	0.188	0.664
Male	287	146	141		
Gender					
<65	217	105	112	0.380	0.537
≥65	317	162	155		
Radiation_therapy_status					
Yes	41	23	18	0.660	0.416
No	493	244	249		
Chemotherapy_status					
Yes	212	98	114	2.003	0.157
No	322	169	153		
TNM_stage					
Stage I	94	59	35	20.828	*P* < 0.001^∗∗∗^
Stage II	197	110	87		
Stage III	163	72	91		
Stage IV	80	26	54		
Invasion_depth					
T1	19	14	5	24.459	*P* < 0.001^∗∗∗^
T2	94	59	35		
T3	365	180	185		
T4	56	14	42		
Lymph_node_metastasis					
Yes	229	90	139	19.062	*P* < 0.001^∗∗∗^
No	303	177	126		
Distant_metastasis					
Yes	80	27	53	10.275	0.001^∗∗^
No	399	213	186		

Bold indicates statistical significance of expression level with ^∗^*P* < 0.05, ^∗∗^*P* < 0.01, and ^∗∗∗^*P* < 0.001. Abbreviations: TCGA: The Cancer Genome Atlas.

**Table 3:** Logistic regression analysis of the association between INHBB expression and clinical characteristics in TCGA cohort.

Clinicopathologic characteristics	TN	OR	95% CI	*P* value
Age (≥65 vs. <65)	534	0.93	0.66-1.32	0.700
Gender (female vs. male)	534	1.17	0.83-1.64	0.364
Invasion depth (T3 & T4 vs. T1 & T2)	534	1.78	1.16-2.75	0.008^∗∗^
Lymph node metastasis (yes vs. no)	532	1.80	1.27-2.55	<0.001^∗∗^
Distant metastasis (yes vs. no)	479	1.76	1.08-2.91	0.026^∗^
TNM stage (stages III & IV vs. stages I & II)	534	1.63	1.16-2.31	0.005^∗∗^

Categorical dependent variable: greater or less than median expression level. OR: odds ratio; TN: total number; CI: confidence interval. Bold indicates statistical significance of expression level with ^∗^*P* < 0.05 and ^∗∗^*P* < 0.01.

**Table 4:** Associations of INHBB expression with overall survival in colorectal cancer.

Parameter	Univariate analysis			Multivariate analysis		
	HR	95% CI	*P* value	HR	95% CI	*P* value
Age (≥65 vs. <65)	2.09	1.31-3.33	0.002	2.71	1.68-4.37	<0.001
Gender (female vs. male)	1.11	0.74-1.68	0.597	—	—	—
Invasion depth (T3 & T4 vs. T1 & T2)	3.88	2.32-6.49	<0.001	2.43	1.40-4.21	0.002
Lymph node metastasis (yes vs. no)	3.23	2.09-6.49	<0.001	0.60	0.21-1.74	0.351
Distant metastasis (yes vs. no)	4.35	2.84-6.67	<0.001	2.07	1.23-3.50	0.006
TNM stage (stages III & IV vs. stages I & II)	3.76	2.39-5.93	<0.001	4.51	1.36-14.90	0.014
INHBB expression (high vs. low)	1.23	1.10-1.37	<0.001	1.13	1.00-1.29	0.039

Abbreviations: TCGA: The Cancer Genome Atlas; HR: hazard ratio; CI: confidence interval.

**Table 5:** Signaling pathways enriched in the INHBB high-expression group.

Signaling pathway	NES	NOM *P* value	FDR *q*-value
KEGG_basal_cell_carcinoma	2.08	≤0.001	0.014
KEGG_vascular_smooth_muscle_contraction	2.04	≤0.001	0.018
KEGG_Hedgehog_signaling_pathway	1.99	≤0.001	0.018
KEGG_ECM_receptor_interaction	1.99	0.002	0.023
KEGG_TGF_beta_signaling_pathway	1.95	≤0.001	0.027
KEGG_focal_adhesion	1.91	0.002	0.034
KEGG_glycosaminoglycan_biosynthesis_chondroitin_sulfate	1.90	≤0.001	0.034
KEGG_arrhythmogenic_right_ventricular_cardiomyopathy_ARVC	1.85	0.002	0.042
KEGG_axon_guidance	1.87	≤0.001	0.042
KEGG_pathways_in_cancer	1.85	≤0.001	0.044
KEGG_melanoma	1.86	0.002	0.046

Note: NOM *P* value < 0.01 and FDR *q*‐value < 0.01 were considered significantly enriched. NES: normalized enrichment score; NOM: nominal; FDR: false discovery rate.

## Data Availability

The data used in this study were downloaded from The Cancer Genome Atlas (TCGA) database and Gene Expression Omnibus (GEO). And the included datasets of the GEO database were GSE23878, GSE44076, GSE25070, GSE44861, GSE38832, and GSE39582. The data used to support the finding of this study are available from corresponding websites upon request.

## Supplementary Materials

Supplementary Materials Supplementary Table 1: the methylation difference of 25 CpG sites between CRC tissue and normal control. Supplementary Table 2: the relationship between INHBB expression and methylation of 25 CpG sites.

## References

[1] Siegel R. L., Miller K. D., Goding Sauer A. (2020). Colorectal cancer statistics, 2020. *CA: a Cancer Journal for Clinicians*.

[2] DeSantis C. E., Lin C. C., Mariotto A. B. (2014). Cancer treatment and survivorship statistics, 2014. *CA: a Cancer Journal for Clinicians*.

[3] Miller K. D., Siegel R. L., Lin C. C. (2016). Cancer treatment and survivorship statistics, 2016. *CA: a Cancer Journal for Clinicians*.

[4] Dienstmann R., Vermeulen L., Guinney J., Kopetz S., Tejpar S., Tabernero J. (2017). Consensus molecular subtypes and the evolution of precision medicine in colorectal cancer. *Nature Reviews. Cancer*.

[5] Schmoll H. J., Van Cutsem E., Stein A. (2012). ESMO consensus guidelines for management of patients with colon and rectal cancer. A personalized approach to clinical decision making. *Annals of Oncology*.

[6] Risbridger G. P., Schmitt J. F., Robertson D. M. (2001). Activins and inhibins in endocrine and other tumors. *Endocrine Reviews*.

[7] Namwanje M., Brown C. W. (2016). Activins and inhibins: roles in development, physiology, and disease. *Cold Spring Harbor Perspectives in Biology*.

[8] Kita A., Kasamatsu A., Nakashima D. (2017). Activin B regulates adhesion, invasiveness, and migratory activities in oral cancer: a potential biomarker for metastasis. *Journal of Cancer*.

[9] Wijayarathna R., de Kretser D. M. (2016). Activins in reproductive biology and beyond. *Human Reproduction Update*.

[10] Tamminen J. A., Yin M., Ronty M. (2015). Overexpression of activin-A and -B in malignant mesothelioma - attenuated Smad3 signaling responses and ERK activation promote cell migration and invasive growth. *Experimental Cell Research*.

[11] Subramanian A., Tamayo P., Mootha V. K. (2005). Gene set enrichment analysis: a knowledge-based approach for interpreting genome-wide expression profiles. *Proceedings of the National Academy of Sciences of the United States of America*.

[12] Liberzon A., Birger C., Thorvaldsdottir H., Ghandi M., Mesirov J. P., Tamayo P. (2015). The Molecular Signatures Database hallmark gene set collection. *Cell Systems*.

[13] Liberzon A., Subramanian A., Pinchback R., Thorvaldsdottir H., Tamayo P., Mesirov J. P. (2011). Molecular Signatures Database (MSigDB) 3.0. *Bioinformatics*.

[14] Mohn F., Weber M., Rebhan M. (2008). Lineage-specific polycomb targets and de novo DNA methylation define restriction and potential of neuronal progenitors. *Molecular Cell*.

[15] Xiong S., Klausen C., Cheng J. C., Zhu H., Leung P. C. (2015). Activin B induces human endometrial cancer cell adhesion, migration and invasion by up-regulating integrin *β*3 via SMAD2/3 signaling. *Oncotarget*.

[16] Worbs S., Shabani N., Mayr D. (2007). Expression of the inhibin/activin subunits (-alpha, -betaA and -betaB) in normal and carcinogenic endometrial tissue: possible immunohistochemical differentiation markers. *Oncology Reports*.

[17] Mylonas I. (2010). Inhibin-alpha, -betaA and -betaB subunits in uterine non-endometrioid carcinomas: prognostic significance and clinical implications. *European Journal of Cancer*.

[18] Watson S. K., Woolcock B. W., Fee J. N. (2009). Minimum altered regions in early prostate cancer progression identified by high resolution whole genome tiling path BAC array comparative hybridization. *Prostate*.

[19] Hofland J., van Weerden W. M., Steenbergen J., Dits N. F., Jenster G., de Jong F. H. (2012). Activin A stimulates *AKR1C3* expression and growth in human prostate cancer. *Endocrinology*.

[20] Wacker I., Behrens J. (2014). Activin B antagonizes RhoA signaling to stimulate mesenchymal morphology and invasiveness of clear cell renal cell carcinomas. *PLoS One*.

[21] Matsuo S. E., Ebina K. N., Kulcsar M. A., Friguglietti C. U., Kimura E. T. (2003). Activin *β*B expression in rat experimental goiter and human thyroid tumors. *Thyroid*.

[22] Mayor R., Casadome L., Azuara D. (2009). Long-range epigenetic silencing at 2q14.2 affects most human colorectal cancers and may have application as a non-invasive biomarker of disease. *British Journal of Cancer*.

[23] Karpinski P., Ramsey D., Grzebieniak Z., Sasiadek M. M., Blin N. (2008). The CpG island methylator phenotype correlates with long-range epigenetic silencing in colorectal cancer. *Molecular Cancer Research*.

[24] Baylin S. B., Jones P. A. (2011). A decade of exploring the cancer epigenome - biological and translational implications. *Nature Reviews. Cancer*.

[25] Jiang L., Hermeking H. (2017). *miR-34a* and *miR-34b/c* suppress intestinal tumorigenesis. *Cancer Research*.

[26] Buhrmann C., Shayan P., Goel A., Shakibaei M. (2017). Resveratrol regulates colorectal cancer cell invasion by modulation of focal adhesion molecules. *Nutrients*.

[27] Beraud-Dufour S., Devader C., Massa F., Roulot M., Coppola T., Mazella J. (2016). Focal adhesion kinase-dependent role of the soluble form of neurotensin receptor-3/sortilin in colorectal cancer cell dissociation. *International Journal of Molecular Sciences*.

[28] Rogers M. A., Kalter V., Marcias G., Zapatka M., Barbus S., Lichter P. (2016). CITED_4_ gene silencing in colorectal cancer cells modulates adherens/tight junction gene expression and reduces cell proliferation. *Journal of Cancer Research and Clinical Oncology*.

[29] Wu C., Zhu X., Liu W., Ruan T., Tao K. (2017). Hedgehog signaling pathway in colorectal cancer: function, mechanism, and therapy. *Oncotargets and Therapy*.

[30] van den Brink G. R., Bleuming S. A., Hardwick J. C. (2004). Indian Hedgehog is an antagonist of Wnt signaling in colonic epithelial cell differentiation. *Nature Genetics*.

[31] Wei L., Lin J., Xu W. (2012). Scutellaria barbata D. Don inhibits tumor angiogenesis via suppression of Hedgehog pathway in a mouse model of colorectal cancer. *International Journal of Molecular Sciences*.

[32] Zhao M., Mishra L., Deng C. X. (2018). The role of TGF-*β*/SMAD4 signaling in cancer. *International Journal of Biological Sciences*.

[33] Calon A., Espinet E., Palomo-Ponce S. (2012). Dependency of colorectal cancer on a TGF-*β*-driven program in stromal cells for metastasis initiation. *Cancer Cell*.

[34] Wiese K. E., Walz S., von Eyss B. (2013). The role of MIZ-1 in MYC-dependent tumorigenesis. *Cold Spring Harbor Perspectives in Medicine*.

