# Supplementary material for: INHBB Is a Novel Prognostic Biomarker Associated with Cancer-Promoting Pathways in Colorectal Cancer
Source: Biomed Res Int. 2020 Oct 6;2020:6909672. doi: 10.1155/2020/6909672 (PMC7563060; doi:10.1155/2020/6909672)
Supplement: Supplementary Materials — Supplementary Table 1: the methylation difference of 25 CpG sites between CRC tissue and normal control. Supplementary Table 2: the relationship between INHBB expression and methylation of 25 CpG sites. [file 6909672.f1.pdf]

Supplementary Table 1 The methylation difference of 25 CpG sites between CRC tissue and normal control.

| CpG sites | normal(βvalue) | tumor(βvalue) | log2(fold change) | *P*_value |
|---|---|---|---|---|
| cg20925841 | 0.041362883 | 0.427422513 | 3.369254249 | 7.29E-11 |
| cg02173749 | 0.035781908 | 0.336779487 | 3.234502044 | 1.02E-09 |
| cg19089337 | 0.022291539 | 0.131736361 | 2.563085475 | 0.02 |
| cg24009722 | 0.051862481 | 0.264345691 | 2.349662681 | 7.10E-09 |
| cg27476576 | 0.01385588 | 0.069097062 | 2.318126028 | 0.007 |
| cg04439159 | 0.03942053 | 0.184057578 | 2.223138071 | 0.00005818 |
| cg07197823 | 0.020665693 | 0.081894672 | 1.986531851 | 0.495 |
| cg03699182 | 0.141575407 | 0.492014369 | 1.797129772 | 2.65E-13 |
| cg07736658 | 0.072513836 | 0.241176289 | 1.733759877 | 1.70E-11 |
| cg24617696 | 0.10793421 | 0.351625428 | 1.703887201 | 8.18E-09 |
| cg23397015 | 0.090336255 | 0.244397718 | 1.435853803 | 0.009 |
| cg06342317 | 0.158959681 | 0.336003502 | 1.079815387 | 2.70E-07 |
| cg26035105 | 0.13865016 | 0.224358618 | 0.69435732 | 0.027 |
| cg03175975 | 0.399867191 | 0.503305312 | 0.331912911 | 0.00009473 |
| cg09174690 | 0.705983191 | 0.747367164 | 0.082183345 | 0.00001117 |
| cg08065733 | 0.451959988 | 0.469076979 | 0.053629643 | 0.221 |
| cg20495333 | 0.793327824 | 0.822009171 | 0.051237341 | 0.000001577 |
| cg14105566 | 0.666836067 | 0.686711543 | 0.042372077 | 0.008 |
| cg03077533 | 0.842043509 | 0.840717261 | -0.002274087 | 0.28 |
| cg14551952 | 0.977975393 | 0.964569775 | -0.019912562 | 0.189 |
| cg23323827 | 0.92141707 | 0.896070933 | -0.040241385 | 0.017 |
| cg13377839 | 0.879817862 | 0.801361015 | -0.134752564 | 1.89E-07 |
| cg25501422 | 0.507801149 | 0.433316973 | -0.228840911 | 6.06E-09 |
| cg00421221 | 0.216174848 | 0.177825508 | -0.28173639 | 3.02E-04 |
| cg09932405 | 0.784990926 | 0.606336335 | -0.372557698 | 4.33E-12 |

Supplementary Table 2 The relationship between INHBB expression and 25 CpG sites methylation.

| CpG sites | Correlation | *P*_value |
|---|---|---|
| cg20925841 | -0.278 | 7.29E-11 |
| cg02173749 | -0.21 | 1.02E-09 |
| cg19089337 | -0.233 | 0.02 |
| cg24009722 | -0.189 | 7.10E-09 |
| cg27476576 | -0.207 | 0.007 |
| cg04439159 | -0.194 | 0.00005818 |
| cg07197823 | -0.22 | 0.495 |
| cg03699182 | -0.304 | 2.65E-13 |
| cg07736658 | -0.235 | 1.70E-11 |
| cg24617696 | -0.289 | 8.18E-09 |
| cg23397015 | -0.336 | 0.009 |
| cg06342317 | -0.221 | 2.70E-07 |
| cg26035105 | -0.064 | 0.027 |
| cg03175975 | -0.198 | 0.00009473 |
| cg09174690 | 0.057 | 0.00001117 |
| cg08065733 | 0.088 | 0.221 |
| cg20495333 | 0.158 | 0.000001577 |
| cg14105566 | 0.227 | 0.008 |
| cg03077533 | -0.003 | 0.28 |
| cg14551952 | 0.064 | 0.189 |
| cg23323827 | 0.11 | 0.017 |
| cg13377839 | 0.218 | 1.89E-07 |
| cg25501422 | 0.237 | 6.06E-09 |
| cg00421221 | 0.093 | 3.02E-04 |
| cg09932405 | 0.163 | 4.33E-12 |
